# The Use of Diatomite-Based Composites for the Immobilization of Toxic Heavy Metals in Industrial Wastes Using Post-Flotation Sediment as an Example

**DOI:** 10.3390/ma17246174

**Published:** 2024-12-17

**Authors:** Krzysztof Gondek, Agnieszka Baran, Patrycja Boguta, Małgorzata Bołdak

**Affiliations:** 1 Department of Agricultural and Environmental Chemistry, University of Agriculture in Krakow, al. Mickiewicza 21, 31-120 Krakow, Poland; krzysztof.gondek@urk.edu.pl (K.G.); malgorzata.boldak@urk.edu.pl (M.B.); 2 Institute of Agrophysics, Polish Academy of Sciences, Doświadczalna 4 Str., 20-290 Lublin, Poland; p.boguta@ipan.lublin.pl

**Keywords:** metals, immobilization, chemical modifications of diatomite, sorption properties, ecotoxicity

## Abstract

Composite materials based on diatomite (DT) with the addition of biochar (BC), dolomite (DL), and bentonite (BN) were developed. The effect of chemical modification on the chemical structure of the resulting composites was investigated, and their influence on heavy metal immobilization and the ecotoxicity of post-flotation sediments was evaluated. It was demonstrated that the chemical modifications resulted in notable alterations to the chemical properties of the composites compared to pure DT and mixtures of DT with BC, DL, and BN. An increase in negative charge was observed in all variants. The addition of BC introduced valuable chemically and thermally resistant organic components into the composite. Among the chemical modifications, composites with the addition of perlite exhibited the lowest values of negative surface charge, which was attributed to the dissolution and transformation of silicon compounds and traces of kaolinite during their initial etching with sodium hydroxide. The materials exhibited varying efficiencies in metal immobilization, which is determined by both the type of DT additive and the type of chemical modification applied. The greatest efficacy in reducing the mobility of heavy metals was observed in the PFS with the addition of DT and BC without modification and with the addition of DT and BC after the modification of H_2_SO_4_ and H_2_O_2_: Cd 8% and 6%; Cr 71% and 69%; Cu 12% and 14%; Ni 10% and Zn 15%; and 4% and 5%. In addition, for Zn and Pb, good efficacy in reducing the content of mobile forms of these elements was observed for DT and DL without appropriate modification: 4% and 20%. The highest reduction in ecotoxicity was observed in the PFS with the addition of DT and BC, followed by BN and DL, which demonstrated comparable efficacy to materials with DT and BN.

## 1. Introduction

The prevention of or reduction in waste generation is a fundamental tenet that is enshrined in numerous regulatory frameworks irrespective of the industrial sector. In certain cases, despite the inability to reduce waste generation, the possibility of reuse should be considered. Waste that is unavoidable or unsuitable for reuse must be disposed of. However, it is imperative to mitigate the potentially deleterious effects of these materials on the environment and human health [1]. The unprotected surface of waste disposal sites represents a significant environmental and human health hazard due to their high susceptibility to blow-off and water erosion [2]. This increases the risk of environmental contamination by pollutants such as dust, odorants, and heavy metals [3,4]. Reducing the potentially negative impact of landfilled waste on the environment and human health is not merely a matter of isolating it from the surrounding environment; rather, it entails the strategic introduction of diverse vegetation. The introduction of vegetation to landfill sites presents a complex challenge requiring the creation of suitable conditions for growth and development. This is particularly difficult due to the defective physical and chemical compositions of the substrate. Vegetation plays a vital role in reducing landfill gas emissions. Plants enhance methane oxidation, achieving an average oxidation capacity twice that of soil without plants, thus lowering greenhouse gas emissions [5]. The presence of plants also decreases heavy metals and nutrient levels in leachate, significantly reducing groundwater pollution risks [6]. The introduction of vegetation fosters biodiversity, with studies showing an increase in plant species over time, which is essential for restoring degraded ecosystems. Vegetation aids in soil stabilization and erosion control, further enhancing the ecological integrity of landfills [7].

The reduction in heavy metal toxicity in the environment through the use of organic and inorganic materials has recently emerged as topic of considerable interest [8,9,10,11,12]. Despite the growing knowledge about the use of diverse materials, the search for the most environmentally friendly solutions persists. This is evident in the promotion of substances, minerals, and composites derived from natural materials. Moreover, the success of immobilization processes or the purification of the environment from active forms of heavy metals depends on the efficiency of the material employed.

Diatomites can be used as an adsorbent material due to their excellent properties, such as their large specific surface area, large pore structure, stable pore wall structure, strong adsorption capacity, mechanical stability, and good hydrothermal stability [13,14]. Adsorption involves the accumulation of atoms, molecules, or ions on the surface of a solid phase. It is a mass transfer process that results in the sorption of gasses or solutes by solid or liquid surfaces [15]. Adsorption onto a solid adsorbent includes three major steps: transportation of the pollutant to the adsorbent surface from an aqueous solution, adsorption onto the solid surface, and transport within the adsorbent particle. Generally, electrostatic attraction causes charged pollutants to adsorb on differently charged adsorbents because heavy metals have a vigorous affinity for hydroxyl (OH^−^) or other functional group surfaces [16,17]. Adsorption is mainly classified into two types: physical adsorption and chemisorption (described as activated adsorption as well). Physical adsorption is the adhesion of an adsorbent to the surface of an adsorbate because of the nonspecific (i.e., independent of the nature of the material) van der Waals force, whereas chemisorption occurs while chemical bonding creates strong attractive forces, i.e., chemical adsorption constructs ionic or covalent bonds through chemical reactions. Nevertheless, physical adsorption is a reversible process but less specific, whereas chemisorption is irreversible but more specific [17,18]. A review of the literature revealed that the properties of diatomites, including those that have undergone chemical modification, and their applications in the environment have been extensively documented [13,19,20]. A well-known routine in the preparation of adsorbents is modifying the adsorbent surface using several methods. These methods include a mechanical process, a thermal process to create pores or a chemical process to improve the surface area, and the use of different radiations.

A chemical technique involves the use of acid, alkali, or salt to improve surface functional groups, and the physical process improves physical properties such as density and solubility. The radiation method proceeds through the formation of free radicals from solvents (H_2_O), polymer backbones, and monomers. The effect of radiation on raw materials is dependent on the energy and frequency range of the applied dose as well as the types of polymers, solvents, and monomers [21]. Surface modification can also be performed using a combination of methods, such as mechanochemical and thermochemical processes [22,23]. The available literature lacks reports on the properties and adsorption efficiencies of diatomite in combination with other mineral and organic materials after chemical modification. The process of chemical modification of the designed mixtures of different materials can result in the formation of novel chemical structures. This can be achieved, for example, by activating functional groups. The activation of initially inactive functional groups will increase the negative surface charge, thereby facilitating the rapid incorporation of a contaminant, such as metal ions, into the composite. This process may also enhance the degree of adsorption of the metal ion [13,24,25].

The objectives of this study were threefold: (i) to develop low-cost composite materials based on diatomite with the addition of biochar, dolomite, and bentonite; (ii) to determine the effect of chemical modification and the addition of perlite to the designed mixtures on the chemical structure of the obtained composites; and (iii) to determine the effect of the obtained composites on the immobilization of heavy metals and the ecotoxicity of the post-flotation sediment. It is noteworthy that this project may represent a response to the current needs of waste management.

## 2. Material and Methods

### 2.1. Selection of Diatomite

The test material was diatomite supplied by Specjalistyczne Przedsiębiorstwo Górnicze “Górtech” Sp. z o. o. The diatomite was obtained from a deposit located in Bircza, Poland (49°43′16.9″ N; 22°18′25.4″ E). The diatomite was subjected to a calcination process. A 0.5 kg sample of diatomite was placed in a chamber furnace where it was exposed to a temperature of 750 °C. The sample was calcined for 0.5 h. This study encompassed a range of grain fractions with a diameter in the range of 0–1 mm.

### 2.2. Materials Used as Additives

The mixtures were prepared using three materials: biochar (BC), dolomite (DL), and bentonite (BN). Biochar (BC) was produced from coniferous biomass by pyrolysis at 500 °C by CarbonTeam. The material had a low ash content (9.29%), an alkaline pH of 7.74, and the largest specific surface area (S_BET_ = 185.6 m^2^/g) of all the additives tested. Dolomite (DL) was obtained from PPUH “Dolomi” Kopalnia Ząbkowice S.A. The pH of the dolomite was alkaline, and the ash content was significantly higher than that of biochar (96.88%). Furthermore, DL had the lowest specific surface area (S_BET_ = 3.0 m^2^/g) among the selected materials. In addition, commercially available calcium bentonite (BN) was employed in the experiment. The ash content of the BN used was 88.6%, and the S_BET_ value was equal to 36.2 m^2^/g. The selected properties of diatomite (DT) and BC, DL, and BN are shown in Table 1.

### 2.3. Procedure for Preparing Test Mixtures

The procedure used to prepare the test mixtures entailed drying the DTs and the mineral and organic materials at 60 °C for 24 h. The mineral and organic additives (BC, DL, and BN) were ground in a laboratory mill (0–0.2 mm). The materials were then mixed in ratios of 20 g DT + 5 g BC (DT+BC), 20 g DT + 5 g DL (DT+DL), and 20 g DT + 5 g BN (DT+BN). The prepared mixtures were placed in a rotary mixer and stirred at 36 rpm for 24 h. The resulting mixtures constituted the starting material for the modification process. The reference material was DT without any additives.

### 2.4. Procedure for Modifying Mixtures

The composites (DT+BC)_1_, (DT+DL)_1_, and (DT+BN)_1_ were obtained by subjecting 25 g of the sample to 25 cm^3^ of H_2_SO_4_ (96%, d = 1.84 kg/dm) and mixing the solution on an orbital shaker for 24 h. Subsequently, 15 cm^3^ of H_2_O_2_ (30%) was added, and the samples were left on a sand bath maintained at 190 °C for another 24 h. Finally, the samples were dried at 340 °C (Table S1).

The composites (DT+BC)_2_, (DT+DL)_2_, and (DT+BN)_2_ were obtained by subjecting 25 g of the sample to 25 cm^3^ of H_2_SO_4_ (96%, d = 1.84 kg/dm) and mixing the solution on an orbital shaker for 24 h. Subsequently, 15 cm^3^ of H_2_O_2_ (30%) was added, and the samples were left on a sand bath maintained at 190 °C for another 24 h. Finally, the samples were dried at 340 °C; 25 cm^3^ of NaOH (30%) was added to the dried material samples and mixed on an orbital shaker for 24 h. The samples were then left on a sand bath at 190 °C for 24 h. Finally, the samples were dried at 340 °C.

The composites (DT+BC)_3_, (DT+DL)_3_, and (DT+BN)_3_ were prepared according to a procedure identical to that used for the composites (DT+BC)_2_, (DT+DL)_2_, and (DT+BN)_2_, except for the following: after drying the materials, 5 g of perlite, 5 cm^3^ of H_2_SO_4_ (96%, d = 1.84 kg/dm), and 5 cm^3^ of H_2_O_2_ (30%) were added. The samples were mixed on an orbital shaker for 24 h. They were then left on a sand bath at 190 °C for another 24 h and subsequently dried at 340 °C.

The composites (DT+BC)_4_, (DT+DL)_4_, and (DT+BN)_4_ were prepared using a procedure identical to that used for the composites (DT+BC)_3_, (DT+DL)_3_, and (DT+BN)_3_, except for the following: after drying the materials, 5 cm^3^ of NaOH (30%) was added and the samples were mixed on an orbital shaker for 24 h. They were then left on a sand bath at 190 °C for another 24 h and subsequently dried at 340 °C. In the study of the chemical modification of DT mixtures with BC, DL, and BN, expanded perlite (EP180) with a grain diameter in the range of 0–2.50 mm was used. The dry matter content of the perlite was 996 g/kg, and the pH value was 7.62.

### 2.5. The Dry Matter Content, pH, and EC in the Composites

The dry matter content was determined in the composites after drying the sample at 105 °C. The pH was determined potentiometrically in a suspension of the composite and distilled water (composite/water = 1:10), and the electrical conductivity (EC) was determined conductometrically (composite/water = 1:10) [12].

### 2.6. The Negative Surface Charge and the Distribution Function of the Apparent Dissociation Constants

The negative surface charge (Q) and the distribution of the apparent surface dissociation constants (f(pKapp)) were determined by potentiometric titration. Measurements were performed in triplicate using a Titrino 702 SM autotitration unit provided by Metrohm (Herisau, Switzerland). A sample weight of 0.1 g was combined with 25 mL of 0.1 M NaCl, adjusted to a pH of 3.0, and placed on a magnetic stirrer for 24 h. Subsequently, the suspensions were readjusted (if necessary) to pH 3.0 with a small volume of HCl or NaOH and were titrated from pH 3.0 to 9.5 with a 0.1 M solution of NaOH prepared on the basis of 0.1 M of NaCl. The reference titration curve performed for 0.1 M of NaCl was subtracted from the curve of the sample NaCl suspension to obtain the signal for the pure sample. Calculations of Q values at various pH values and f(pKapp) functions for the studied composites were carried out according to the methodology proposed by Szatanik-Kloc et al. [26].

### 2.7. FT-IR Spectroscopic Study on Structure

Samples for FTIR measurements were prepared as a thin pellet in a press (10 t) from a mixture of 1 mg of powdered composite with 200 mg of KBr (spectral purity). The spectra were recorded on a Tensor 27 spectrometer (Bruker Optics, Billerica, MA, USA) in the range of 400–4000 cm^−1^. Characteristics were obtained as the mean of two measurements with 256 scans at 2 cm^−1^ resolution each. The data were processed using the instrument software OPUS (https://www.bruker.com/en/products-and-solutions/infrared-and-raman/opus-spectroscopy-software.html) (Bruker Optics, Billerica, MA, USA), which includes a smoothing function, transmittance to absorbance conversion, and baseline correction.

### 2.8. Incubation Experiment

Incubation tests were conducted under laboratory conditions using 100 mL PVC containers containing 20 g of post-flotation sediment (PFS). The experimental design included 17 treatments, with each treatment performed in triplicate (Table 4). The amount of each composite introduced into the PFS was 5% (*w*/*w*). After introducing the composites into the PFS, the material was wetted with distilled water and thoroughly mixed. It was then placed in PVC containers and transferred to an incubator, where the experiment was conducted for 152 days. During this period, the temperature in the incubator was maintained at 20 °C, and the humidity of the material was kept at 45% of the water capacity. The selected properties of the PFS are shown in Table 2.

### 2.9. Chemical and Ecotoxicological Analyses After Incubation in PFS

After the incubation experiment, the following parameters were determined in the post-flotation sediment with the addition of composites: pH—potentiometrically in a suspension of sediment and distilled water (sediment/water = 1:10); electrical conductivity (EC)—conductometrically (sediment/water = 1:10). The mobilities of metals (Cd, Cr, Cu, Ni, Zn, and Pb) in the test treatments were evaluated using the modified BCR sequential extraction procedure [27,28].

The heavy metal content of the obtained extracts was determined by inductively coupled plasma atomic emission spectrometry (ICP-OES, Perkin Elmer Optima 7300 DV, (PerkinElmer, Inc., Waltham, MA, USA).

The effectiveness of the composites in immobilizing heavy metals in PFS was assessed using three factors: Risk Assessment Code (RAC), Individual Contamination Factor (ICF), and Ecological Risk Factor (ERF) [28]. The RAC factor was calculated as a percentage of the total content accumulated in the F1 fraction of the heavy metals. The F1 fraction accumulates heavy metal forms that are readily exchangeable, water- and acid-soluble, and combined with carbonates. In these combinations, metals are readily activated and thus become readily bioavailable posing a significant ecological risk [29]. The ICF was calculated as the ratio of the heavy metal content of the potentially accessible fraction (∑F1–F3) to the metal content of the residual fraction (F4), while the ERF was calculated as the ratio of the sum of the metal content of the F1 and F2 fractions to the sum of the metal content of the F3 and F4 fractions. A common feature of the calculated ratios is that a smaller value indicates a lower degree of mobility and bioavailability for the heavy metal under study.

The ecotoxicity of PFS and mixtures of PFS with composites was evaluated using two tests: Ostracodtoxkit F and Microtox. In the Ostracodtoxkit F test, the crustacean *Heterocypris incongruens* was used as the test organism [30]. Following a 6-day incubation period of *H. incongruens* with test samples on multi-well plates at 25 °C in a dark environment, the mortality and growth inhibition of the crustaceans were evaluated. The Microtox test employed the luminescent bacteria *Alivibrio fischeri* as the test organisms. The inhibition of luminescence in the prepared extracts (1:10) was quantified before and after 15 min of incubation of the bacteria with the test sample. The Microtox M500 analyzer [31] was used to perform the test. The ecotoxicity of the samples was evaluated according to toxicity classes as follows: class I (PE ≤ 20%, no significant toxic effect, non-toxic sample); class II (20% < PE ≤ 50%, significant toxic effect, low toxic sample); class III (50% < PE < 100%, significant toxic effect, toxic sample)—acute hazard; and class IV (PE = 100%, highly toxic sample).

### 2.10. Statistics

The significance of the differences between the mean parameter values was analyzed using a one-way analysis of variance (ANOVA) and Tukey’s test at a significance level of 0.05. Statistical analyses were performed using Microsoft Excel 2016 and the Statistica 13 software package.

## 3. Results

### 3.1. The Dry Matter (DM) Content and pH and EC Values of the Composites

The dry matter content of the tested composites did not exhibit any significant variation (V% = 7.0) and averaged 940.5 ± 65.6 g/kg (Table 3) irrespective of the additive and modification procedure employed. It was observed that, regardless of the DT additive, there was a consistent decline in the dry matter content of the composites produced according to the method in which H_2_SO_4_ (96%, d = 1.84 kg/dm) and H_2_O_2_ (30%) were introduced into the mixtures. In other composites, the dry matter content was typically higher than that observed in unmodified mixtures.

The modification procedures carried out for the designed DT mixtures with BC, DL, and BN resulted in a notable reduction in the pH values of the composites. The chemical composition of perlite is as follows: 70–75% SiO_2_, 12–15% Al_2_O_3_, 3–4% Na_2_O O, 3–5% K_2_O, 0.5–2% Fe_2_O_3_, 0.2–0.7% MgO, and 0.5–1.5% CaO 3–5%. The indicated content of metal oxides can contribute to reducing the deleterious effects of concentrated sulfuric acid by forming neutral salts (Table 3).

The EC determined in the developed mixtures exceeded 300 µS/cm (Table 3). The modification procedures conducted for the DT-based mixtures reduced this parameter by a factor of three or more irrespective of the addition.

### 3.2. The Negative Surface Charge and the Distribution Function of the Apparent Dissociation Constants

**Figure 1:**
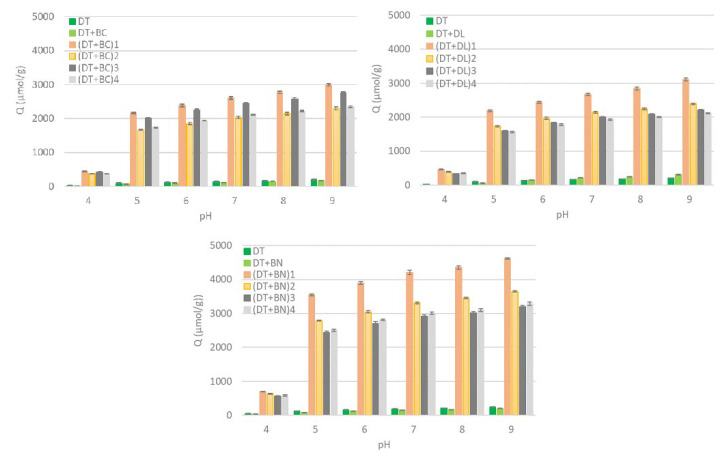
Distribution of surface charge (Q) derived from functional groups at increasing pH values.

**Figure 2:**
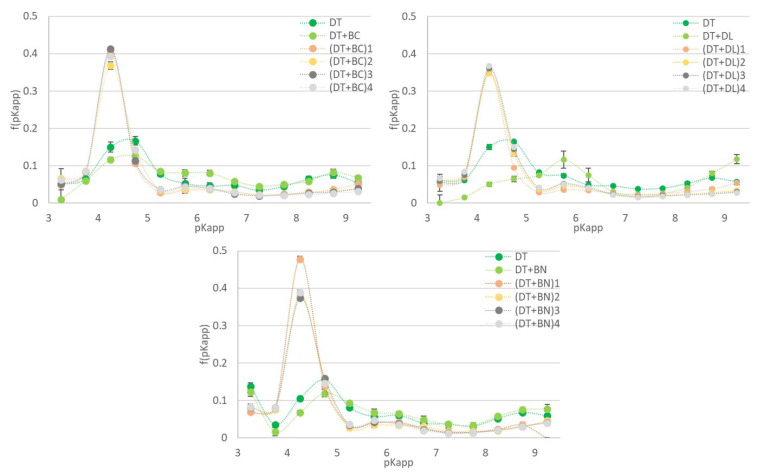
The distribution function of the apparent dissociation constants in a wide range of pK.

The results of the potentiometric titrations are presented in Figure 1 and Figure 2, respectively, in the form of the distribution of surface charge (Q) and the distribution function of the apparent dissociation constants (f(pKapp)) over a wide range of pH values.

The contribution of negative surface charge from the studied materials increased with increasing pH levels resulting from the dissociation of subsequent functional groups during the titration process. At a low pH, protons of stronger acidic groups were released. A further increase in the pH resulted in the removal of protons from moderately acidic and weakly acidic groups, respectively [32]. Among the chemically unmodified materials (DT, DT+BC, DT+DL, and DT+BN), the highest Q value (measured at pH 9) was found for DT combined with DL (304 µmol/g), while the lowest value was noted for DT combined with BC (176 µmol/g).

The chemical–thermal modification of the mixtures resulted in a significant increase in the Q values, probably related to the activating effect of the chemical agents. The greatest increase in this parameter was observed when the pH increased from 4.0 to 5.0. These regions correspond to the dissociation of strongly acidic groups [32]. The Q development at a higher pH was still progressive but less intense, indicating a smaller contribution of less acidic groups [33].

The Q value was the highest for the chemical modification of DT+BN mixtures compared to the modification of DT mixtures with DL and BC irrespective of the modification variant. Within the chemical modification variants, the composites with BC showed the highest Q values for (DT+BC)_1_ and the lowest ones for (DT+BC)_2_ and (DT+BC)_4_. Similarly, the composites with DL exhibited the highest Q values for the (DT+DL)_1_ modification. Interestingly, both types of modifications with perlite resulted in the lowest Q values within the DL- and BN-modified composites.

The distribution function of the apparent dissociation constants demonstrates the proportion of groups that dissociate under specified pH conditions relative to the total pool of functional groups that generate a negative surface charge. In unmodified mate-rials (DT, DT+BC, and DT+BN), the above distribution showed a slight increase in the contribution of groups dissociating at pH 4.25–4.75, and a comparatively weaker increase in the groups dissociating at pH ~ 8.75 (also for DT+DL). Furthermore, an additional pool of functional groups was observed in the case of DT+DL (pK ~ 5.75), which was associated with the presence of carbonates [34].

The chemical modification of each type of mixture resulted in the formation of a dominant pool of structures with higher acidity (with the maximum shifting from ~4.75 to ~4.25). The largest increase in the proportion of this type of structure was obtained for modified BN-based composites, while within the group of various modifications, the variant involving H_2_SO_4_ + H_2_O_2_ treatment demonstrated the largest increase. At the same time, the relative contribution of the other groups decreased.

### 3.3. An FT-IR Spectroscopic Study on the Structure of Diatomite-Based Composites

**Figure 3:**
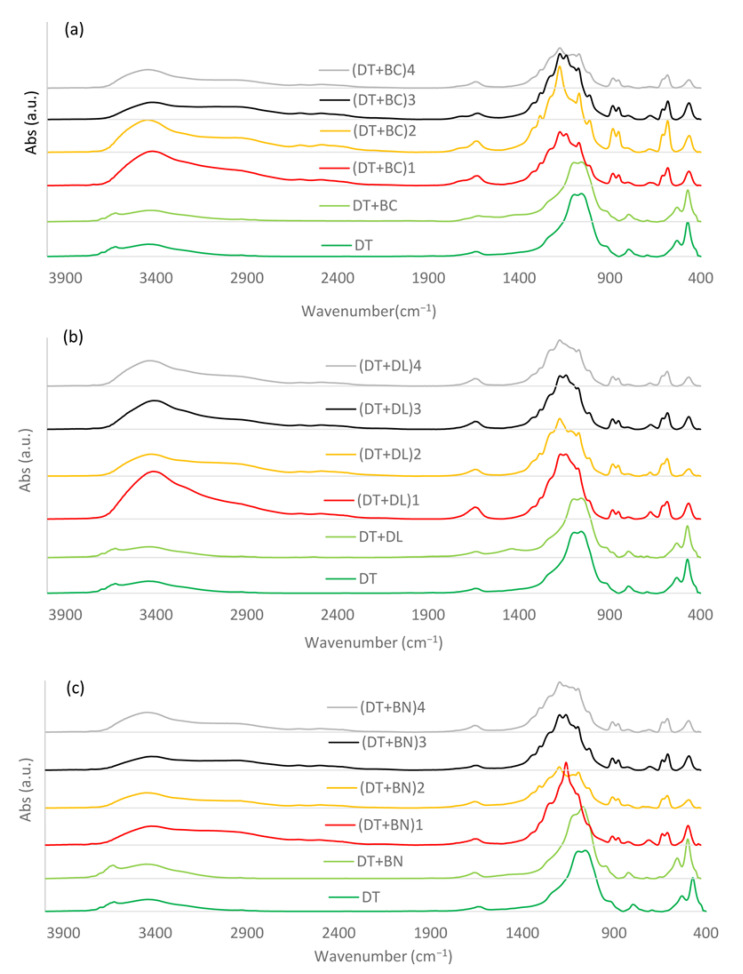
The FI–IR spectra of the diatomite (DT) and DT composites with (**a**) biochar (BC), (**b**) dolomite (DL), and (**c**) bentonite (BN).

The FTIR spectra of the composites tested revealed a number of bands characteristic of the initial components (Figure 3). Bands typical of DT were observed in pure material and in chemically unmodified mixtures with BC, DL, and BN. In this case, absorbance at ~3630 cm^−1^ was attributed to the presence of kaolinite and the stretching vibrations in the outer and inner surface O––H of the octahedral sheets, which form an H-bond with the O of the tetrahedral sheets [35]. The signals at ~3450 cm^−1^ and ~1647 cm^−1^ were related to–OH stretching and H–O–H bending, respectively, in physically adsorbed water [36]. Bands located at ~1103 cm^−1^ and ~1050 cm^−1^ are assigned to vibrations in Si-O apical and Si–O–Si (Al), respectively [37]. Weaker but narrow signals located at ~800 cm^−1^, 520 cm^−1^, and 467 cm^−1^ originate from OH translational vibrations, Si–O–Al symmetric stretching, and Si-O-Si bending vibrations, respectively [37].

The contribution of the BC, DL, and BN additives to the DT spectrum was partially overlapped by DT signals. However, the addition of BC slightly changed the DT spectrum by introducing signals from organic structures such as C=O, C–O, N–H, O–H, C=C, and CH_x_ in the ranges of ~1600–1350 cm^−1^ and ~2900–2800 cm^−1^ [38,39]. The DL addition resulted in a new band at ~1433 cm^−1^ related to the presence of CO_3_^2−^ [40], while the addition of BN enhanced the band at ~3630 cm^−1^, ~3450 cm^−1^, and at ~1050 cm^−1^, which was attributed to vibrations of the bentonite structural units, Al-OH-Al, the OH stretching of structural hydroxyl groups, and water present in the mineral as well as layered silicate montmorillonite mineral [41].

The FTIR spectra of the chemically modified composites showed clear structural changes compared to the physical mixtures and pure DT. The band at ~3630 cm^−1^ disappeared and the band at ~3450 cm^−1^ increased for all modifications, suggesting changes in the OH structures and the possibility of H-bond formation. The enhancement at ~3450 cm^−1^ was the strongest for the composites with DL, especially for the H_2_SO_4_ + H_2_O_2_ treatment. Interestingly, the modification variants (DT+BC)_3_ showed the weakest enhancement at ~3450 cm^−1^. A significant broadening of the signal in the region of 3400–2500 cm^−1^ may confirm the increased proportion of H-bonds. Significant changes were observed in the middle spectral region for the modified compositions based on BC and DL. Modification nos. 1, 2, 3, and 4 caused an increase at 1647 cm^−1^ for BC and DL composites (for BN composites, there was a very weak increase only in modifications 2 and 4), which was attributed to the increase in OH bending vibrations of adsorbed water in phyllosilicate minerals and in BC from C=O of COO^−^. A slight increase at 1740 cm^−1^ was also observed for all modifications of BC composites, suggesting an increase in C=O of carboxylic groups and possibly traces of aldehydes, ketones, and esters [42]. The FTIR spectra of the modified mixtures show a clear reduction in the bands at ~1103 cm^−1^ and ~1050 cm^−1^ related to the presence of Si-O and Al-O structures and the appearance of new sharp signals at ~1180 and 1145 cm^−1^.

### 3.4. Mobility of Heavy Metals

The total content is not a significant parameter in the evaluation of heavy metal immobilization. The most reliable information on the efficiency of the immobilization process, as well as the potential environmental consequences associated with the mobility of metals, is provided by determining the chemical forms of metals [43,44]. As previously stated, the objective of this study was to use composites to immobilize metals in the PFS with the intention of reducing their mobility.

The mobility of the studied metals according to the RAC factor differed significantly not only by the type of composite, but also by the type of element. The share of cadmium accumulated in the F1 fraction in the total content was 28% after the addition of DT to the PFS, representing the lowest level (Table 4). The introduction of DT+BC, DT+DL, and DT+BN mixtures into the PFS without modification resulted in a more pronounced reduction in the proportion of mobile forms (F1) of Cd than composites subjected to chemical modification. In general, cadmium mobility increased when the modified materials were added sequentially to the PFS irrespective of the DT additive used. The highest proportion of cadmium forms accumulated in F1 in the total content was observed in the PFS to which (DT+BC)_4_, (DT+DL)_4_, and (DT+BN)_3_ composites were introduced. According to the RAC classification, treatments in which DT, DT+BC, and (DT+BC)_1_ were added to the PFS showed a moderate risk of Cd release. The remaining study treatments demonstrated a high risk associated with the mobility of this metal.

Similarly to Pb, Cr exhibited a high potential mobility. The highest proportions of Cr and Pb accumulated in F1 were observed in the PFS treatment (Table 4). The incorporation of mixtures and composites into the PFS after chemical modification resulted in a notable reduction in the RAC value compared to the control treatment (PFS). This reduction ranged from 13% to 75% for Cr and from 9% to 20% for Pb. As for Cd, the lowest RAC values were observed in treatments where non-chemically modified mixtures were introduced into the PFS. In general, an inverse relationship was observed for Pb, with lower RAC values being obtained when chemically modified composites were added to the PFS compared to unmodified mixtures. The treatments with DT, DT+BC, (DT+BC)_1_, and DT+DL showed a minimal risk of Cr release. The potential for the release of this metal was identified as medium in mixtures of (DT+BC)_2_, (DT+DL)_1_, and DT+BN. In the remaining treatments, very high and high risks were identified for the mobility of Cr. For Pb, a high potential risk of releasing this metal was determined regardless of the additive. However, it should be noted that the total Cr content in the mixtures analyzed was significantly lower compared to Cd and Pb.

After the addition of Cu to the PFS DT and the other materials tested, the RAC value ranged from 7 to 25% (Table 4). In the control treatment (PFS without additives), the RAC value was determined to be 21%. The most pronounced effect was observed for mixtures of DT with BC, DL, and BN, which had not undergone any chemical modification. The DT+BC mixture and the chemically modified composites derived from it were found to be the most effective in reducing Cu mobility in the PFS. Furthermore, the FTIR spectra and the observed increase in the bands at ~1600 cm^−1^ and ~1700 cm^−1^ indicate the high activity of the developed composites in reducing Cu mobility. The mean reduction in RAC values following the addition of biochar to DT was 9% compared to the control. In contrast, the mean reduction in RAC values following the addition of materials developed on the basis of DT and dolomite and bentonite was only 2.4%. According to the RAC classification, the mixtures of DT, namely DT+BC, (DT+BC)_1_, and DT+DL, exhibited a minimal risk of Cu release. Conversely, the remaining study treatments showed a moderate risk associated with the mobility of this metal.

The RAC value for Ni, like that for Cu, was relatively low (Table 4). The RAC value for Ni ranged from 11% to 28% in the treatments with the addition of the tested materials, while in the control treatment (PFS control), the RAC value was 26%. As for Cd and Cu, a lower mobility of Ni in the PFS was observed after the addition of mixtures without chemical modification. It is also noteworthy that comparable RAC values were obtained in treatments where composites were introduced into the PFS after modification with concentrated H_2_SO_4_ and H_2_O_2_. In the case of the (DT+BC)_1_ composite, these values were even lower than those calculated for the treatments with mixtures without chemical modification. All treatments showed a moderate risk related to Ni mobility.

The RAC values for Zn in the treatments where the tested materials were added to the PFS ranged from 16% to 22%, while the RAC for the control treatment (PFS without additives) was 21% (Table 4). The results demonstrate that the highest efficiency in Zn immobilization was achieved when DT+BC-based materials were added to the PFS. The incorporation of chemical modification and perlite in the composites (except (DT+BC)_1_) resulted in an increase in Zn mobility. Regardless of the addition to the PFS, the risk of release of this metal was at an average level.

The individual contamination factor (ICF) is used to evaluate the capacity of a given heavy metal to be released from samples under different environmental conditions [45]. The introduction of DT-based mixtures or composites into the PFS after chemical modification yielded disparate ICF values (Table 4). In general, the addition of composites to the PFS resulted in increased ICF values for Cd (except for (DT+BC)_1_), Cr, and Zn, indicating an increase in their mobility compared to the PFS without additives (control). The opposite situation was observed for Pb and Cu (with the exception of (DT+DL)_2_, (DT+BN)_3_, and (DT+BN)_4_). In the case of Ni, a reduction in its mobility based on the ICF was observed after the application of DT+BC, DT+DL, and DT+BN mixtures to the PFS that had not undergone any chemical modification. Such a reduction was also observed following the application of (DT+BC)_1_, (DT + DL)_1_, and (DT+BN)_1_ composites to the PFS modified with concentrated H_2_SO_4_ and H_2_O_2_. Since the ICF reflects the risk of heavy metal contamination associated with the potential mobility of metals, the analyzed samples showed significant contamination for Pb (all treatments) and Cr ((DT+BC)_4_, (DT+DL)_3_, (DT+DL)_4_, and (DT+BN)_4_). Moderate contamination rates were recorded for Ni (except for (DT+BC)_1_), Cd (except for (DT+BC)_1_ and (DT+DL)_3_), and Cr (DT+BC, (DT+BC)_2_, (DT+BC)_3_, (DT+DL)_1_, (DT+BN)_1_, and (DT+BN)_2_). Low mobility-related contamination rates were identified for Zn (in all treatments), Cu (except for (DT+DL)_2_, (DT+BN)_2_), Cd ((DT+BC)_1_, and (DT+DL)_3_), and Cr ((DT+BC)_1_, DT+DL, (DT+DL)_4_, and DT+BN).

The above observations were partially confirmed by the ERF. It was demonstrated that the incorporation of composites into the post-flotation sediments generally reduced the factor value for Pb, Ni, and Zn compared to the treatment with the PFS alone (Table 4). Lower ERF values indicate an increase in the metal content of the less mobile F3 (organic matter-bound) and F4 (residual) fractions, thereby reducing the risk of their release into the environment. This relationship was also confirmed for Cd (DT, DT+BC, (DT+BC)_1_, DT+DL, (DT+DL)_1_, (DT+DL)_2_, (DT+DL)_3_, DT+BN, (DT+BN)_1_, and (DT+BN)_2_) and Cu (DT, DT+BC, (DT+BC)_1_, (DT+BC)_2_, (DT+BC)_3_, (DT+BC)_4_, DT+DL, (DT+DL)_1_, (DT+BN)_1_, (DT+BN)_2_, and (DT+BN)_4_). This relationship was not shown for Cr in most mixtures (except DT+BC, (DT+BC)_1_, (DT+BC)_2_, DT+DL, and DT+BN). The probability of Cu and Zn mobility was low in the samples tested. With regard to Cd and Ni, the mixtures exhibited a medium risk of release into the environment. In the case of Pb (all treatments) and Cr ((DT+BC)_3_, (DT+BC)_4_, (DT+DL)_2_, (DT+DL)_3_,) (DT+DL)_4_, (DT+BN)_2_, (DT+BN)_3_, and (DT+BN)_4_), a high risk was identified for their potential mobility to the environment (Table 4). It is also noteworthy that, irrespective of the metal type, the chemical modification of the composites and the addition of perlite resulted in enhanced metal mobility in the PFS compared to mixtures without chemical modification. The average values of the ERE factor indicate that the highest effectiveness in reducing the mobility of metals in the PFS was observed following the addition of DT mixtures with BC (except Cr), while the lowest effectiveness was determined for materials developed on the basis of DT and BN (except Cu).

The pH values determined in each treatment were generally at similar or significantly higher levels compared to the pH value determined in the PFS control treatment (Table 5). Irrespective of the addition of DT and the chemical modification, the enrichment of the PFS with the developed materials resulted in an elevated pH value compared to the pH value determined in the PFS control. The observed increase in pH value may have been due to the activation of the alkaline ion charge as a result of chemical modification, as indicated by the determined EC values (Table 5). The effectiveness in triggering the alkaline ion charge from the developed materials is confirmed by the lower EC value determined in the treatments where DT+BC, DT+DL, and DT+BN mixtures that were not chemically modified were used as additives to the PFS, which fell to a level comparable to that of the PFS control treatment.

### 3.5. Ecotoxicity of PFS After Composite Application

The test results show a notable decline in the ecotoxicity of the PFS enriched with the developed materials compared to the PFS control (Table 5). The inhibition of *A. fischeri* luminescence exhibited a considerable range of sensitivity across individual treatments, with values spanning from −18 to 28%. In contrast, the PFS control treatment demonstrated a significantly higher inhibition rate of 42%. The greatest reduction in toxicity to the bacteria was observed in the following treatments: PFS + (DT+BC)_1_, PFS + (DT+BC)_2_, PFS + DT+DL, PFS + (DT+DL)_2_, and PFS + (DT+BN)_2_. These treatments exhibited stimulation of luminescence. It is noteworthy that the majority of the tested samples showed non-toxicity to *A. fischeri*, with a low toxicity of 26% observed only in the PFS + (DT+BN)_1_ and PFS + (DT+BN)_2_ treatments. The invertebrate *H. incogurens* demonstrated a higher sensitivity to the substances present in the PFS enriched with the developed materials than *A. fischeri*. Ostracod mortality in the experimental treatments ranged from 0% to 60%, while growth inhibition ranged from 26% to 59%. In the PFS control treatment, both parameters reached 100%. Significantly, the lowest toxicity to *H. incongruens* was observed in the PFS + (DT+BC)_2_ treatment, followed by PFS + (DT+BC)_3_, PFS + (DT+BC)_4_, PFS + (DT+DL)_3_, and PFS + (DT+BN)_3_. The results show low toxicity to *H. incongruens* in these treatments. In contrast, the remaining treatments exhibited toxicity to the ostracod, while the PFS control was found to be highly toxic. The greater sensitivity of *H. incongruens* compared to *A. fischeri* may be attributed to a number of factors. First, the primary route of exposure to toxic substances for this organism is the oral route [46]. Additionally, the sensitivity of the test organisms may be influenced by the method of exposure to toxic substances. In the Ostracodtoxkit test, organisms are exposed to a range of substances, including soluble compounds and contaminants adsorbed on particulate mixtures. In contrast, the Microtox test uses *A. fischeri* and focuses on bioavailable compounds that are readily soluble in water. The post-flotation sediment from the control treatment was classified as class IV, indicating a high level of toxicity. Class II comprised samples of low acute risk to organisms, including PFS enriched with biochar-based materials such as (DT+BC)_2_, (DT+BC)_3_, (DT+BC)_4_, dolomite (DT+DL)_3_, and bentonite (DT+BN)_3_. The remaining PFS samples were classified as toxicity class III, indicating a high acute risk (Table 5). Conversely, the averaged results for individual mixtures demonstrate that the most effective materials in reducing ecotoxicity for test organisms were those formulated on the basis of DT and BC, followed by BN and DL, which performed similarly to those formulated on the basis of DT and BN.

## 4. Discussion

The search for innovative and effective methods to reduce environmental pollution is becoming a pivotal issue in the context of the growing phenomenon of anthropopresion. Soil, a key component of terrestrial ecosystems, often becomes a repository for pollutants, including heavy metals, as a result of industrial activities, agricultural practices, and inadequate waste management. The presence of heavy metals in the environment represents a significant threat to both ecological and human health. These metals can enter the food chain through uptake by crops, thereby posing a potential risk to human well-being. In this context, in the present study, the authors (i) developed composite materials based on diatomite, biochar, dolomite, and bentonite; (ii) investigated the surface charge and distribution functions of the apparent dissociation constants and FTIR spectra; and (iii) tested the prepared composites for their effects on heavy metal mobility and the ecotoxicity of zinc and lead ore flotation sludges. A discussion of the obtained results was challenging due to the very limited number of studies in this area.

The chemical modification of natural materials is one of the promising approaches to develop new, efficient conditioners suitable for remediation purposes. The components used in the formation of the composites studied are highly susceptible to chemical modification. DT, with its hydroxyl groups and structured porosity, can serve as an interesting basis for modified composite materials used in sorption applications. Our study has shown that pure DT has a relatively low negative surface charge. This result was similar to the previously reported CEC values of various diatomite samples in the range of 11–28 cmol/kg [47]. The application value of such unmodified material is mainly due to its porosity, which determines the filtration and structural properties [48]. Physically mixing DT with other components such as DL, BN, and BC only results in additivity of the sorption properties of individual components, but no modification of DT should be expected in this case. For such mixtures, we obtained the best Q results for the DT+DL mixture (304 μmol/g, at pH 9) due to the increased contribution of mono- and bi-charged carbonate ions in higher pH ranges [33]. For such conditions, the presence of carbonates can result in the product having improved buffering capacity and pH stability. We also observed carbonates’ buffering activity in the physical mixture of DT+DL when there was a higher pool of carbonates on the distribution function of dissociation constants at pK~5.75. However, it should be mentioned that at pH values that are more common for different types of applications, such as soil or water treatment, these mixtures did not show an increased Q value. Moreover, this parameter was often lower than that for pure DT because some of the functional groups of the additives generated negative charges at higher pH levels. In the above case, the modification of the material was fully justified.

The chemical modification of DT increased the Q value of the composites, indicating significant changes in the chemical and structural properties of the compounds. Interestingly, the highest Q increase was determined for the initial mixtures of (DT+DL), (DT+BN), and (DT+BC) after modification with H_2_O_2_-H_2_SO_4_ solutions. These findings indicate the activating effect of strong oxidants. According to Boriskov et al. [49], acid activation could lead to the dissolution of metal oxide-releasing micro- and nanopores and result in increased sorption activity in diatomite. Steudel et al. [50] reported that clay minerals such as bentonite treated with mineral acids (H_2_SO_4_ and HCl) dissolved the octahedral sheets by interlayer and edge attack, and that the substituted cations promoted these reactions and the formation of a silica phase. Similar acid modification combined with heating was reported to be favorable for the activation of bentonite. It improved the textural properties of the clay, resulting in better dispersibility in water and increased surface area, mesopore volume, number of surface adsorption sites, and siloxane groups, but it decreased the number of hydroxyl groups in bentonite [51]. On the other hand, there are also studies showing that the crystalline structures of DT were stable under acid treatment [52]. However, it should be noted that in most of the above studies, the activation process involved the removal of the reactive solution. In our study, it was heated and dried at a high temperature along with the solid residue, and as a result, dissolved and chemically transformed components were not removed from the composite mass. In addition, there was also an excess of acid and its salts in the system, which could significantly affect the amount of titrant consumed and, ultimately, the Q value. Only in the case of DL could chemically modified composite carbonates be removed from acidic solutions as gaseous by-products, resulting in probable reductions in the buffering capacity and pH stability of the final material.

The lowest Q values were found in perlite-supplemented combinations modified with both H_2_SO_4_-H_2_O_2_ and NaOH (exception: (DT+BC)_3_ variant). It showed that perlite—particularly with a combination of alkaline modifiers, was not the most optimal choice for chemical modifications aimed at increasing the number of negatively dissociating functional groups. Interestingly, as mentioned above, the composites based on BC and perlite from variant no. 3 (without the modification of perlite with NaOH) did not show such a significant decrease in the Q values as the composites based on DL and BN. This may indicate an indirect modification resulting from the interaction of perlite with the mass of composite under the environment of H_2_SO_4_ and H_2_O_2_. Considering the deteriorated properties after modification with NaOH, it should be emphasized that previous studies conducted on perlite showed that modification with NaOH can lead to the dissolution and transformation of SiO_2_ [53]. A similar mechanism may also occur in mixtures without perlite but modified with NaOH, as silicic acid and biogenic Si from diatom frustules are unstable at a high pH [46]. According to Boriskov et al. [49], the dissolution of silicon may release an additional amount of hydroxyl groups, increasing the number of active sorption sites. Furthermore, alkali treatment could also degrade traces of kaolinite [52]. Our findings are consistent with the above conclusions since each variant with NaOH digestion had a Q value lower than the corresponding variant without NaOH. The dissolution of SiO_2_ in variant 4 can therefore be a continuation of the same process as that in variant 2. This is also confirmed by the lower Q values of variant 4 compared to variant 2 for all samples, as well as the reduced FTIR bands at ~1050 cm^−1^ and ~455 cm^−1^ derived from Si––O asymmetric stretching and Si–O–Si or Al–O–Al bending vibrations, respectively. The kaolinite traces of DT observed in the FTIR spectra at ~3600 cm^−1^ also disappeared under the chemical modifications. Furthermore, the appearance of new absorption bands at ~600 cm^−1^, ~700 cm^−1^, and ~900 cm^−1^ for NaOH-modified materials (especially with perlite) may indicate the formation of zeolite-like materials [53]. Previous investigations have shown that NaOH-modified perlite has strong sorption properties with respect to Eu^3+^ and Ce^3+^ ions, but in these studies, the post-reaction solution was rejected [54].

Chemical modifications can improve Q, resulting in higher sorption capacities of the composite. The increased sorption of heavy metals may result from an increase in the number of functional groups that generate a negative surface charge, such as OH or COOH [55]. However, the higher charge indicated by the potentiometric measurement may also include the determination of negatively charged sulfuric acid ions. Chemical changes in all modified compounds could also occur under the influence of high temperatures (compounds marked with factors 1–4 were dried at 340 °C). The thermogravimetric study by Reka et al. [35] showed that the weight loss occurring at 265 °C was due to the elimination of adsorbed and absorbed water in DT. The second temperature interval, between 265 and 600 °C, was attributed to the dehydration process of the chemically bound water in the opal structure and the combustion of the organic matter present in DT. Even higher temperatures (not used in our study) caused the dehydroxylation of clay components (muscovite and kaolinite).

However, it should be mentioned that the sorption of toxic substances (immobili-zation) can also be improved by changing the surface characteristics, i.e., porosity, sur-face area, or hydrophilic/hydrophobic properties of the surface. Among other things, the modification of diatomaceous earth by alkaline etching led to the preparation of mesoporous adsorbents with a high surface area; however, silicon dioxide in the diatomite sample was reported to be unreactive during acid treatment [56]. Guo et al. [57] found a significant improvement in the adsorption capabilities of diatomite pretreated with NaOH due to the generation of SiO_3_^2−^ species with a larger specific surface area. For this reason, a decrease in the Q value, e.g., in the case of composites modified with perlite and acids/bases/H_2_O_2_, does not necessarily mean a given contaminant has a weaker sorption efficiency.

As reported by Samani et al. [58], the application of modified diatomaceous earth reduced the concentrations of Pb, Zn, Cu, Cr, and Ni extracted with DTPA in comparison to unmodified treatments. As stated by the aforementioned authors, the use of a 10% addition of modified diatomite provided sufficient surface areas for the adsorption of heavy metals, thereby reducing their concentration in the soil solution. In our own study, we employed a variety of additional modifications beyond the use of chemicals (H_2_SO_4_, NaOH, and H_2_O_2_) alone. These included the incorporation of biochar, dolomite, bentonite, or perlite, which significantly impacted the activity of the resulting composites in terms of heavy metal immobilization, as evidenced by the values of the RAC, ICF, and ERF. The modification procedures could activate hydroxyl groups on the surface of the composites, which are characterized by a high adsorption capacity and occur in the form of free silanol groups (Si-OH), free silanol-diol groups (Si-(OH)_2_), and atomic bridges with oxygen ions (Si-O-Si) [59]. The efficiency of the immobilization of individual elements by the composites showed variability. In certain instances, superior immobilization outcomes were observed when DT+BC, DT+DL, and DT+BN mixtures without chemical modification were employed in conjunction with PFS. On the one hand, the absorption capabilities of the composites may have been related to the hydration radius and electronegativity of the ion. However, the relatively low addition of the composites (5% *w*/*w*) to the PFS may have been a limiting factor. In the case of some heavy metals, it is likely that the lower hydration radius and higher electronegativity are the reason for their greater absorption [2]. A study by Samani et al. [58] showed that modified diatomite significantly reduced the bioaccumulation of heavy metals in contaminated soils with the exception of Zn.

In the present study, the addition of the developed composites affected the pH of the PFS (Table 5). The developed DT- and BC-based composites caused the greatest increase in pH values in the PFS. Samani et al. [58] also showed a significant effect of modified diatomite on the soil pH, which, according to the cited authors, is an important factor in reducing the mobility of heavy metals in soil.

Krawczynska et al. [60] investigated the effect of organic matter on the mobility of heavy metals in post-flotation sediments. According to the cited authors, the introduction of pulp from sugar beet processing into the PFS increased copper leaching and increased the toxicity of the extracts. On the other hand, Ciarkowska et al. [4] showed a positive effect of the addition of sewage sludge to the PFS, which, among other things, led to a decrease in the content of available zinc. However, it should be noted that when organic materials are added to the PFS, there is a process of mineralization, which may result in the degradation of organic compounds containing heavy metals in their combinations. Such a possibility is confirmed by the results obtained by Gondek et al. [4]. The study conducted by Mierzwa-Hersztek et al. [61] on soils artificially contaminated with Zn, Pb, and Cd revealed that the mechanism of the process of reducing the mobility of ions of the studied heavy metals may be related to changes in the properties of the substrate, including the redox potential, pH, and the introduction into the soil of materials with significantly developed sorption surfaces capable of effectively binding metal ions. The cited authors concluded that the adsorbents used (zeolite and biochar) have the potential to sorb heavy metals from contaminated soils. The degree of immobilization depends on the type of metal, its concentration in solution, and the amount and type of adsorbent used. It should also be noted that time can be an important factor modifying the efficiency of heavy metal immobilization when such materials are used.

The findings of numerous authors indicate that post-flotation sediments are not an optimal medium for organism growth [4,62,63,64]. The reduction in the toxicity of post-flotation sediments may be attributed to the fact that the additives used improved the physical and chemical properties of the sediment, which may have led to improved air and water conditions, particularly for *H. incongruens* [65]. From an ecotoxicological perspective, the toxicity of the PFS enriched with the developed composites was not attributable to its pH. The physical properties (size and porosity) and the relatively high EC value may have played more significant roles in the observed effects on the test organisms used [64,66].

## 5. Conclusions

The experimental data obtained and the results of the incubation test show that the developed diatomite-based materials have great environmental potential in terms of managing the bioavailability of heavy metals and reducing the ecotoxicity of landfilled wastes.

Qualitative analyses showed that the chemical modifications resulted in significant changes in the chemical properties of the composites compared to pure DT and mixtures of DT with BN, BC, and DL. An increase in the negative charge was observed in all the variants studied, which may indicate an increased sorption capacity of the activated materials. In particular, modifications with acid and hydrogen peroxide led to an improvement in this parameter. The addition of BC introduced valuable chemically and thermally resistant organic components into the composite. Among the chemical modifications, the composites with perlite showed the lowest values of negative charge, which resulted from the dissolution and transformation of silicon compounds and traces of kaolinite during their previous etching with sodium hydroxide. The reduction in the negative surface charge could be related to the reduced immobilization of metals chemically bonding through negatively charged carboxyl and phenolic groups. However, the effectiveness of impurity immobilization in this case did not have to correlate positively with the value of the negative charge because modifications with acid, and especially with alkali, could lead to surface activation, i.e., an increase in porosity and the specific surface area. These changes could increase the immobilization capacity of the composites by adsorption. Such changes in the skeleton of silicate compounds were also confirmed by FTIR spectra.

The calculated heavy metal mobility factors, RAC, ICF, and ERF, indicate that the developed materials exhibit varying efficiencies in immobilizing heavy metals, which is determined by both the type of diatomite additive used and the type of chemical modification applied. The analysis of the pH and EC values of the materials from the incubation experiment revealed that, regardless of the DT addition and chemical modification, the enrichment of PFS with the developed materials increased the pH of this material. This was achieved by triggering the alkaline ion charge as a result of the chemical modification, as indicated by the EC values obtained. The averaged results for individual mixtures indicate that the materials developed on the basis of DT and BC exhibited the greatest effectiveness in reducing ecotoxicity to test organisms (*A. fischeri* and *H. incongruens*). This was followed by BN and DL, which showed comparable effectiveness to materials developed on the basis of DT and BN.

Further research is required to target the functionalization of the developed materials with respect to a specific heavy metal, the type of substrate (waste), and the interaction of the materials in the substrate (waste)–composite–plant system.

## Figures and Tables

**Table 1:** Selected physical and chemical properties of the materials used in the experiment.

Material *	Dry Matter g/kg	Ash g/kg dm	pH H_2_O	EC ** µS/cm	BET Surface Area m^2^/g	Total Pore Volume cm/g
DT	855 ± 3	808 ± 3	5.84 ± 0.05	392 ± 1	25.9 ± 0.9	0.064 ± 0.002
BC	952 ± 4	99 ± 0	7.74 ± 0.02	330 ± 2	185.6 ± 6.6	0.088 ± 0.003
DL	942 ± 4	915 ± 3	7.87 ± 0.04	107 ± 1	3.0 ± 0.1	0.009 ± 0.000
BN	949 ± 4	881 ± 3	9.31 ± 0.01	842 ± 8	36.2 ± 1.3	0.115 ± 0.004
mg/kg dm	Cd	Cr	Cu	Ni	Pb	Zn
DT	0.34 ± 0.06	29.68 ± 0.50	45.3 ± 0.8	21.67 ± 0.73	14.06 ± 0.08	40.7 ± 1.2
BC	4.95 ± 0.01	42.57 ± 1.33	14.4 ± 2.3	18.77 ± 3.02	2.26 ± 0.40	30.0 ± 1.3
DL	1.89 ± 0.19	2.21 ± 0.19	13.2 ± 0.7	1.61 ± 0.15	11.40 ± 0.07	38.8 ± 1.9
BN	1.06 ± 0.03	4.96 ± 0.41	21.6 ± 2.1	1.13 ± 0.03	14.56 ± 0.05	28.6 ± 2.3

Each value represents the mean of three replicates ± SE. * DT—diatomite; BC—biochar; DL—dolomite; BN—bentonite; ** EC—electrical conductivity.

**Table 2:** Selected physical and chemical properties of PFS.

Granulometric Composition (%)			pH H_2_O	EC mS/cm	C g/kg dm
1–0.1 mm	0.1–0.02 mm	<0.02 mm			
70	21	9	7.37 ± 0.13	0.76 ± 0.02	2.61 ± 0.01
Cd	Cr	Cu	Ni	Pb	Zn
mg/kg dm					
69.6 ± 1.9	3.30 ± 0.70	15.81 ± 2.23	14.15 ± 1.14	7,500 ± 29	10,200 ± 48

Each value represents the mean of three replicates ± SE.

**Table 3:** The dry matter (DM) content and pH and EC values of the composite materials.

Material/Composite	DM * g/kg	pH	EC ** µS/cm
DT+BC	952.4 ± 0.6	6.26 ± 2.83	380.6 ± 1.2
(DT+BC)_1_	817.4 ± 0.1	0.80 ± 11.56	75.2 ± 4.4
(DT+BC)_2_	995.1 ± 0.4	0.62 ± 8.05	90.2 ± 5.2
(DT+BC)_3_	968.1 ± 0.6	0.58 ± 2.44	77.0 ± 4.2
(DT+BC)_4_	954.2 ± 0.1	0.65 ± 1.10	62.8 ± 10.1
DT+DL	964.1 ± 0.1	6.93 ± 1.43	360.9 ± 3.6
(DT+DL)_1_	805.4 ± 0.4	0.75 ± 3.77	65.6 ± 3.0
(DT+DL)_2_	993.7 ± 0.3	0.57 ± 2.48	74.5 ± 4.1
(DT+DL)_3_	981.8 ± 0.1	0.49 ± 7.29	88.0 ± 0.1
(DT+DL)_4_	952.4 ± 0.0	0.59 ± 3.63	70.5 ± 4.1
DT+BN	938.0 ± 0.7	7.42 ± 1.62	358.4 ± 2.0
(DT+BN)_1_	900.0 ± 0.8	0.75 ± 0.00	54.8 ± 3.6
(DT+BN)_2_	992.0 ± 0.1	0.58 ± 12.19	61.5 ± 1.6
(DT+BN)_3_	963.1 ± 0.2	0.55 ± 7.71	94.9 ± 4.8
(DT+BN)_4_	962.2 ± 0.1	0.70 ± 5.09	60.5 ± 2.4

Each value represents the mean of three replicates ± SE. * DM—dry matter; ** EC—electrical conductivity.

**Table 4:** Heavy metal mobility parameters in PFS after incubation.

Treatment	Cd	Cr	Cu	Ni	Pb	Zn
	RAC (% in Total Metals Bound to F1) ^1^					
PFS (Control)	38 cd ^4^	85 c	21 cd	26 cd	69 d	21 ab
PFS + DT	28 a	13 a	7 a	17 bc	61 bcd	16 a
PFS + DT+BC	30 abc	14 a	9 a	16 ab	60 bcd	17 a
PFS + (DT+BC)_1_	32 bc	16 a	7 a	11 a	52 ab	16 a
PFS + (DT+BC)_2_	39 cd	36 ab	13 abc	22 bcd	53 ab	22 b
PFS + (DT+BC)_3_	38 cd	71 cd	15 bcd	28 e	60 bcd	22 b
PFS + (DT+BC)_4_	40 d	50 abc	16 bcd	21 bcd	55 abc	22 b
PFS + DT+DL	32 bc	10 a	9 a	15 ab	62 bcd	17 a
PFS + (DT+DL)_1_	35 bc	25 ab	13 abc	17 bc	60 bcd	20 ab
PFS + (DT+DL)_2_	35 bc	43 abc	25 d	20 bcd	49 a	20 ab
PFS + (DT+DL)_3_	37 bcd	58 abc	22 cd	27 e	57 abc	21 ab
PFS + (DT+DL)_4_	38 cd	70 cd	23 cd	26 cd	54 abc	20 ab
PFS + DT+BN	32 bc	12 a	11 ab	15 ab	64 cd	18 ab
PFS + (DT+BN)_1_	36 bc	51 abc	16 bcd	19 bcd	59 bc	21 ab
PFS + (DT+BN)_2_	37 bcd	47 abc	25 d	25 cd	56 abc	20 ab
PFS + (DT+BN)_3_	39 cd	49 abc	23 cd	26 cd	54 abc	21 ab
PFS + (DT+BN)_4_	37 bcd	72 cd	19 bcd	25 cd	58 bc	21 ab
	**ICF (∑F1−-F3)/F4 ^2^**					
PFS (Control)	1.00 ab	0.00 a	0.90 cde	1.43 abc	4.49 b	0.63 a
PFS + DT	1.00 ab	1.73 a	0.39 ab	1.24 abc	3.68 ab	0.81 ab
PFS + DT+BC	1.17 ab	2.68 ab	0.33 a	1.28 abc	2.98 ab	0.98 ab
PFS + (DT+BC)_1_	0.79 a	0.53 a	0.32 a	0.60 a	2.82 a	0.72 ab
PFS + (DT+BC)_2_	1.26 ab	1.60 a	0.56 abc	1.49 abc	3.14 ab	1.00 b
PFS + (DT+BC)_3_	1.08 ab	1.70 a	0.74 bcd	1.91 bc	3.85 ab	0.78 ab
PFS + (DT+BC)_4_	1.28 b	3.26 ab	0.77 bcd	2.00 bc	4.19 ab	0.93 ab
PFS + DT+DL	1.07 ab	0.95 a	0.48 abc	1.08 ab	3.37 ab	0.81 ab
PFS + (DT+DL)_1_	1.15 ab	2.12 a	0.50 abc	1.36 abc	3.76 ab	1.00 b
PFS + (DT+DL)_2_	1.02 ab	6.78 b	1.16 e	1.87 bc	3.97 ab	0.87 ab
PFS + (DT+DL)_3_	0.99 ab	3.93 ab	0.83 cde	1.70 bc	4.20 ab	0.69 ab
PFS + (DT+DL)_4_	1.10 ab	0.94 a	0.95 cde	2.24 b	4.36 ab	0.79 ab
PFS + DT+BN	1.15 ab	0.97 a	0.45 abc	1.03 ab	3.27 ab	0.96 ab
PFS + (DT+BN)_1_	1.03 ab	1.73 a	0.40 ab	1.27 abc	3.63 ab	0.80 ab
PFS + (DT+BN)_2_	1.04 ab	1.92 a	1.07 de	1.82 bc	4.12 ab	0.76 ab
PFS + (DT+BN)_3_	1.13 ab	0.92 a	0.85 cde	2.04 bc	4.14 ab	0.83 ab
PFS + (DT+BN)_4_	1.00 ab	4.24 ab	0.84 cde	2.03 bc	4.33 ab	0.73 ab
	**ERF (F1 + F2)/(F3 + F4) ^3^**					
PFS (Control)	0.73 bc	0.40 ab	0.31 abcd	0.51 f	3.32 d	0.32 ab
PFS + DT	0.49 a	0.19 a	0.13 ab	0.25 ab	2.31 abc	0.23 a
PFS + DT+BC	0.52 ab	0.21 a	0.13 ab	0.23 ab	2.18 ab	0.23 a
PFS + (DT+BC)_1_	0.48 a	0.15 a	0.11 a	0.14 a	2.01 a	0.24 ab
PFS + (DT+BC)_2_	0.78 bc	0.64 a	0.22 abc	0.32 bcde	2.39 abcd	0.33 ab
PFS + (DT+BC)_3_	0.74 bc	6.27 c	0.21 abc	0.47 f	2.72 abcd	0.33 ab
PFS + (DT+BC)_4_	0.81 c	1.30 a	0.22 abc	0.33 bcde	2.83 abcd	0.34 b
PFS + DT+DL	0.56 abc	0.14 a	0.14 abc	0.22 ab	2.31 abc	0.24 ab
PFS + (DT+DL)_1_	0.64 abc	0.43 ab	0.20 abc	0.25 ab	2.63 abcd	0.29 ab
PFS + (DT+DL)_2_	0.64 abc	1.41 a	0.47 d	0.30 bcd	2.58 abcd	0.29 ab
PFS + (DT+DL)_3_	0.71 bc	3.60 bc	0.31 abcd	0.46 f	3.07 cd	0.30 ab
PFS + (DT+DL)_4_	0.74 bc	2.65 bc	0.35 bcd	0.41 def	2.98 bcd	0.30 ab
PFS + DT+BN	0.56 bc	0.14 a	0.25 abcd	0.22 ab	2.52 abcd	0.25 ab
PFS + (DT+BN)_1_	0.67 bc	0.79 ab	0.18 abc	0.29 bc	2.71 abcd	0.31 ab
PFS + (DT+BN)_2_	0.71 bc	1.39 ab	0.37 cd	0.40 cdef	2.82 abcd	0.30 ab
PFS + (DT+BN)_3_	0.75 bc	2.50 b	0.34 bcd	0.42 ef	2.90 bcd	0.32 ab
PFS + (DT+BN)_4_	0.69 bc	4.08 bc	0.26 abcd	0.40 cdef	3.09 cd	0.31 ab

^1^ RAC ≤ 1% no risk, 1% < RAC ≤ 10% low risk, 10% < RAC ≤ 30% medium risk, 30% < RAC ≤ 50% high risk, 50% < RAC very high risk; ^2^ ICF ≤ 1 low contamination, 1 < ICF ≤ 3 moderate contamination, 3 < ICF ≤ 6 considerable contamination, ICF > 6 very high contamination; ^3^ 0 < ERF ≤ 0.4 low risk, 0.4 < ERF ≤ 1 medium risk, 1 < ERF high risk. ^4^ The same letter (a, b, c, or d) means no significant differences between the values at the level of significance (α = 0.05), one-way ANOVA, and Tukey’s HSD test.

**Table 5:** The pH, EC, and ecotoxicity of the PFS after incubation based on the responses of test organisms and classifications of their toxicity.

Treatment	pH	EC	*A. fischeri*	*H. Incongruens* Pb		Toxicity Class ^4^
		mS^2^cm^−1^	IL% ^3^	M % ^1^	GI % ^2^	
PFS control	7.24 a ^5^	1.07 a	42 c	100 d	100 b	IV
PFS + DT	7.49 ab	0.97 a	2 ab	67 bcd	50 a	III
PFS + DT+BC	7.63 ab	0.86 a	8 ab	60 bcd	67 ab	III
PFS + (DT+BC)_1_	7.59 ab	1.94 b	−18 a	70 cd	59 ab	III
PFS + (DT+BC)_2_	7.74 b	2.52 ef	−15 a	0 a	26 a	II
PFS + (DT+BC)_3_	7.80 b	2.01 bc	5 ab	10 ab	35 a	II
PFS + (DT+BC)_4_	7.82 b	2.48 ef	5 ab	30 abc	40 a	II
PFS + DT+DL	7.60 ab	0.97 a	−1 ab	83 cd	72 ab	III
PFS + (DT+DL)_1_	7.72 b	2.04 bc	0 ab	50 bcd	53 ab	III
PFS + (DT+DL)_2_	7.47 ab	2.69 f	−10 a	70 cd	34 a	III
PFS + (DT+DL)_3_	7.61 ab	2.20 cd	0 ab	47 bcd	39 a	II
PFS + (DT+DL)_4_	7.75 b	2.51 ef	2 ab	67 bcd	50 a	III
PFS + DT+BN	7.54 ab	1.06 a	28 b	73 cd	72 ab	III
PFS + (DT+BN)_1_	7.65 ab	1.92 b	26 b	50 bcd	38 a	II
PFS + (DT+BN)_2_	7.76 b	2.68 f	−5 ab	63 bcd	37 a	III
PFS + (DT+BN)_3_	7.63 ab	2.09 bc	0 ab	47 bcd	41 a	II
PFS + (DT+BN)_4_	7.83 b	2.33 de	13 ab	67 bcd	44 a	III

^1^ M—*Heterocypris incongruens*, mortality; ^2^ GI—*Heterocypris incongruens*, growth inhibition; ^3^ AfLI—*Allivibrio fischeri*, luminescence inhibition. ^4^ Toxicity classes: class I (PE ≤ 20%, non-toxic sample); class II (20% < PE ≤ 50%, low toxic sample); class III (50% < PE < 100%, toxic sample); class IV (PE = 100% highly toxic sample). ^5^ The same letter (a, b, c, or d) means no significant differences between the values at the level of significance (α = 0.05), one-way ANOVA, and Tukey’s HSD test.

## Data Availability

Data are contained within the article and Supplementary Materials. Further inquiries can be directed to the corresponding author.

## Supplementary Materials

The following supporting information can be downloaded at: https://www.mdpi.com/article/10.3390/ma17246174/s1, Table S1: The combination and treated method of diatomite and additives.
