# Supplementary material for: The Use of Diatomite-Based Composites for the Immobilization of Toxic Heavy Metals in Industrial Wastes Using Post-Flotation Sediment as an Example
Source: Materials (Basel). 2024 Dec 17;17(24):6174. doi: 10.3390/ma17246174 (PMC11676275; doi:10.3390/ma17246174)
Supplement: Supplementary file 1 [file materials-17-06174-s001.zip › materials-3332499-supplementary.pdf]

Table S1: The combination and treated method of diatomite and additives

Designation	Treatment
DT	
DT+BC	DT (20 g) + BC (5 g)
$(DT+BC)_1$	DT (20 g) + BC (5 g) + H_2SO_4 (25 cm^3) + H_2O_2 (15 cm^3)
$(DT+BC)_2$	DT (20 g) + BC (5 g) + H_2SO_4 (25 cm^3) + H_2O_2 (15 cm^3) + NaOH (25 cm^3)
$(DT+BC)_3$	DT (20 g) + BC (5 g) + H_2SO_4 (25 cm^3) + H_2O_2 (15 cm^3) + NaOH (25 cm^3) + Perlite (5 g) + H_2SO_4 (5 cm^3) + H_2O_2 (5 cm^3)
$(DT+BC)_4$	DT (20 g) + BC (5 g) + H2SO4 (25 cm^3) + H_2O_2 (15 cm^3) + NaOH (25 cm^3) + Perlite (5 g) + H_2O_2 (5 cm^3) + H_2SO_4 (5 cm^3) + NaOH (5 cm^3)
DT+DL	DT (20 g) + DL (5 g)
$(DT+DL)_1$	DT (20 g) + DL (5 g) + H_2SO_4 (25 cm^3) + H_2O_2 + (15 cm^3)
$(DT+DL)_2$	DT (20 g) + DL (5 g) + H_2SO_4 (25 cm^3) + H_2O_2 (15 cm^3) + NaOH (25 cm^3)
$(DT+DL)_3$	DT (20 g) + DL (5 g) + H_2SO_4 (25 cm^3) + H_2O_2 (15 cm^3) + NaOH (25 cm^3) + Perlite (5 g) + H_2SO_4 (5 cm^3) + H_2O_2 (5 cm^3)
$(DT+DL)_4$	DT (20 g) + DL (5 g) + H_2SO_4 (25 cm^3) + H_2O_2 (15 cm^3) + NaOH (25 cm^3) + Perlite (5 g) + H_2O_2 (5 cm^3) + H_2SO_4 (5 cm^3) + NaOH (5 cm^3)
DT+BN	DT (20 g) + BN (5 g)
$(DT+BN)_1$	DT (20 g) + BN (5 g) + H_2SO_4 (25 cm^3) + H_2O_2 (15 cm^3)
$(DT+BN)_2$	DT (20 g) + BN (5 g) + H_2SO_4 (25 cm^3) + H_2O_2 (15 cm^3) + NaOH (25 cm^3)
$(DT+BN)_3$	DT (20 g) + BN (5 g) + H_2SO_4 (25 cm^3) + H_2O_2 (5 cm^3) + NaOH (25 cm^3) + Perlite (5 g) + H_2SO_4 (5 cm^3) + H_2O_2 (5 cm^3)
$(DT+BN)_4$	DT (20 g) + BN (5 g) + H_2SO_4 (25 cm^3) + H_2O_2 (5 cm3) + NaOH (25 cm^3) + Perlite (5 g) + H_2O_2 (5 cm^3) + H_2SO_4 (5 cm^3) + NaOH (5 cm^3)
